# Remember, recognize, revere: spaces for the memory of the technical staff at Fiocruz

**DOI:** 10.1590/S0104-59702023000100071

**Published:** 2023-12-15

**Authors:** Renata Reis

**Affiliations:** i Professora e pesquisadora, Escola Politécnica de Saúde Joaquim Venâncio / Fiocruz . Rio de Janeiro – RJ – Brasil renata.reis@fiocruz.br

**Keywords:** Technical workers, Instituto Oswaldo Cruz, Memory, Heritage education, Trabalhadores técnicos, Instituto Oswaldo Cruz, Memória, Educação patrimonial

## Abstract

This article reflects on the justifications for the “Lugares de Memória: história e vida dos trabalhadores técnicos da Fiocruz” project, a heritage education initiative linking the architectural heritage of the Manguinhos campus to the memory and many histories of the laboratory assistants who worked at the Oswaldo Cruz Institute (Fiocruz) during its first thirty years of existence. The objective was to establish a space for memory for the institution’s technical staff by creating a dialog between the past and present in the history of Fiocruz and integrating virtual spaces and historical environments.

## Resumo

O artigo reflete sobre as justificativas do projeto “Lugar de memória: história e vida dos trabalhadores técnicos da Fiocruz”, uma iniciativa de educação patrimonial que relaciona o patrimônio arquitetônico do *campus* Manguinhos com a memória e as muitas histórias dos auxiliares de laboratório que atuaram no Instituto Oswaldo Cruz em seus trinta primeiros anos de existência. O propósito do projeto foi estabelecer um lugar de memória para os trabalhadores técnicos da instituição, compondo um diálogo entre passado e presente da história da Fiocruz e integrando espaços virtuais a ambientes históricos.

> Era a nossa casa. Aqui comíamos, aqui dormíamos. Isto aqui era uma maravilha, compreende? (Attilio Borriello, 13 jun. 1986).

Este artigo tem como propósito descrever o projeto “Lugar de memória: história e vida dos trabalhadores técnicos da Fiocruz”, um projeto de educação patrimonial que relaciona o patrimônio arquitetônico do *campus* Manguinhos/Fiocruz com a memória e as muitas histórias dos auxiliares de laboratório que atuaram no Instituto Oswaldo Cruz, atual Fiocruz, em seus trinta primeiros anos de existência. Os resultados previstos pelo projeto compreendem uma exposição virtual de longa duração, que se conecta diretamente com placas instaladas em pontos estratégicos do Núcleo Arquitetônico e Histórico de Manguinhos (Nahm), contendo informações que associam seus monumentos arquitetônicos com as histórias dos auxiliares de laboratório que ali viviam ou trabalhavam.

Essa iniciativa, além de contribuir para o registro da história e preservação da memória desse grupo de trabalhadores da Fiocruz, pode colaborar na criação de novos equipamentos para compor as áreas de visitação pedagógica do *campus* que poderão ser utilizadas pelo público visitante do Museu da Vida, pelos estudantes da Escola Politécnica de Saúde Joaquim Venâncio (EPSJV) e de outras unidades de ensino da Fiocruz, por escolas de ensino fundamental e médio, e por toda a comunidade da Fiocruz que circula pelo *campus*. A iniciativa visa favorecer a democratização do conhecimento sobre a história da instituição, a ressignificação da memória institucional, o estímulo a novas pesquisas e investigações sobre o tema e a integração entre ciência, cultura e sociedade por meio da história.

O artigo foi organizado de forma a apresentar alguns desses trabalhadores, revelando seus rostos mediante fotografias que compõem o arquivo histórico da Fiocruz e arquivos familiares, buscando refletir sobre o papel dos técnicos na produção da atividade científica na instituição, para, em seguida, apresentar o projeto em si, que busca relacionar educação patrimonial, história, memória e divulgação do conhecimento científico.

Os trabalhadores técnicos representam hoje mais de 80% do contingente total de trabalhadores em saúde no Brasil, distribuídos em diversas categorias profissionais desde cuidadores de idosos, agentes comunitários de saúde e auxiliares de enfermagem até tecnólogos de nível superior.

Sua atuação envolve diferentes modalidades de assistência à saúde, da atenção primária até a alta complexidade hospitalar, estando presentes também nas equipes de estudos e pesquisas voltadas para a produção de medicamentos, vacinas, insumos, complementação diagnóstica e outros.

Nos últimos tempos, diante da pandemia da covid-19, esses trabalhadores foram fundamentais no cuidado com os doentes, nos processos de pesquisa que envolvem medicamentos e vacinas, no apoio diagnóstico de exames laboratoriais de confirmação e no monitoramento da evolução da doença.

Historicamente, no entanto, os trabalhadores técnicos em saúde têm experimentado políticas de qualificação que não atendem a suas demandas específicas por formação, reconhecimento e regulamentação profissional, impactando trajetórias de vida e trabalho. Expostos aos processos de precarização, que envolvem formas de contratação, direitos, condições de trabalho, incluindo relações sociais determinadas por uma hierarquização de saberes e subordinação de suas práticas, a eles fica reservado um lugar de menor valor dentro das equipes, instituições onde atuam e junto à sociedade (Batistella, 2013; Morosini, 2010; Vieira, Durão, Lopes, 2011; Vieira, Chinelli, 2013).

Os reflexos desse cenário se fazem sentir também no campo da história, tendo em vista a pouca produção de estudos que se ocuparam do trabalho e dos trabalhadores técnicos da saúde. Com exceção do artigo de Jaime Benchimol (1989) publicado no final dos anos 1980 "Retratos do cotidiano em Manguinhos", reimpresso recentemente (Benchimol, 2022), há pouca informação. O livro de Olympio da Fonseca Filho (1974) e as duas publicações de Carlos Chagas Filho (1993, 2000) fazem menção aos auxiliares de laboratório com base em suas memórias pessoais sobre a convivência com esses trabalhadores.

Diante da vasta historiografia sobre a gênese da Fundação Oswaldo Cruz e seus célebres personagens, todos conhecemos, ou pelo menos já ouvimos falar, em Oswaldo Cruz, Carlos Chagas, Adolpho Lutz, Henrique Aragão, Ezequiel Dias, Cardoso Fontes e outros, com suas presenças imortalizadas em livros, em fundos e coleções arquivísticas, na nomenclatura de edificações, ruas, praças e monumentos do *campus* da instituição.

Os marcos citados acima configuram "lugares de memória" no sentido utilizado por Pierre Nora (1993), que nos faz refletir sobre a relação entre o valor do passado e o sentimento de pertencimento a um determinado grupo, situando-se entre a memória individual e a memória coletiva. Nessa perspectiva, os lugares de memória têm também estreita relação com a constituição de uma identidade comum.

Em sua origem, no entanto, quando ainda era denominada Instituto Oswaldo Cruz, a Fiocruz não foi construída somente pelos cientistas. Junto deles estavam também o que hoje seriam os trabalhadores técnicos daquele período: os auxiliares de laboratório.

Esses trabalhadores atuaram lado a lado com os cientistas nas pesquisas experimentais, na produção de medicamentos, vacinas, soros e outros insumos, no trabalho de campo das expedições científicas, no ensino, e tiveram uma participação fundamental no desenvolvimento da ciência e da saúde pública brasileira, mas são praticamente desconhecidos pela comunidade da Fiocruz.

Joaquim Venâncio, apesar de homenageado na ocasião de fundação de uma unidade técnico-científica que leva seu nome – a Escola Politécnica de Saúde Joaquim Venâncio, criada em agosto de 1985 –, não possuía uma biografia sistematizada. Até a realização da pesquisa que fundamentou este projeto, o conhecimento sobre ele limitava-se a uma fotografia e a alguns artigos de pesquisadores (Ferreira, 2003; Paraense, 1988) baseados em suas lembranças e em "causos" que se contavam, recriando memórias sobre esse personagem.

Não é objetivo deste artigo discutir essas lacunas e silêncios, tampouco aprofundar uma análise sobre o processo histórico de produção e organização da ciência que estabeleceu a separação e a hierarquização entre o saber fazer dos técnicos e dos cientistas. Entretanto, o apagamento dos trabalhadores técnicos nos processos de construção do fazer científico, e mesmo das equipes de saúde em que atuam, persiste até os dias atuais.

Bruno Latour (1997), ao analisar a produção social do objeto científico, observou que a distinção é utilizada pelos cientistas quando buscam construir suas carreiras individuais, que não estão necessariamente atreladas às questões materiais e econômicas. Já os técnicos, ao contrário, dependem totalmente do elemento material para consolidar suas carreiras. Ao analisar os grupos e equipes de pesquisa, observa que as múltiplas trajetórias de seus profissionais acabam convergindo para uma "hierarquia de posições administrativa quase perfeita" (Latour, 1997, p.244).

Para o autor, essa hierarquia pode estar relacionada ao papel que cada indivíduo desempenha no processo de produção do conhecimento, à autoria dos artigos científicos e à facilidade com que determinado membro da equipe pode ser substituído, variando de acordo com a complexidade do trabalho desenvolvido.

Historicamente, o ambiente da intelectualidade e da ciência, tradicionalmente, pertencia àqueles considerados "cavalheiros".

Steven Shapin (2013), ao estudar a gênese da Royal Society, instituição secular do Reino Unido dedicada a promover a excelência na ciência, afirma que a condição de cavalheiro era o que assegurava a credibilidade do conhecimento experimental.

> Mais importante é que a forma das relações sociais de uma assembleia composta de homens não livres, ou pior, uma sociedade dividida entre livres e não livres, corromperia o processo pelo qual o conhecimento experimental deveria ser construído e avaliado, e pelo qual esse conhecimento podia ser anunciado como sendo confiável. Homens não livres eram aqueles a quem faltava o controle arbitrário de suas próprias ações. Os técnicos, por exemplo, pertenciam a essa classe – a classe dos empregados – porque seu trabalho científico braçal era pago (Shapin, 2013, p.81).

No Brasil, a organização da ciência acompanhou e reiterou essa distinção, especialmente nas décadas iniciais da Primeira República. Os cientistas eram, em sua maioria, descendentes de famílias administradoras e proprietárias de terras, letrados, considerados cavalheiros. Seus auxiliares, os descendentes daqueles que se empregavam no trabalho na terra, homens pobres, peões, colonos, capatazes, feitores e suas ferramentas. Para esses era imprescindível não só empregar sua força de trabalho, como também provar que eram "homens de bem", acima de qualquer suspeita, esforçados, dedicados.

O legado da escravidão e os costumes de uma sociedade colonial eram marcas recentes e, por isso mesmo, encontravam-se fortemente presentes nas relações sociais vigentes.

## Caminhos da memória

O conceito de memória está relacionado com lembranças de acontecimentos que envolvem afetos. É um processo vivido, conduzido por grupos vivos, e por isso mesmo em permanente evolução, vulnerável a muitas manipulações (D'Alessio, 1992-1993).

Para a memória não existe passado e presente, pois se reporta à herança, à tradição. Ao mesmo tempo, é o que move nossa existência social, pois é a única maneira de sobrepor a vida à morte, o espírito contra o vazio, renovando os elos entre gerações e a preocupação com o passado coletivo (Petersen, 2013).

Para Nora (1993, p.9), é preciso que tomemos consciência de que memória e história não são sinônimos: "A história é a reconstrução sempre problemática e incompleta do que não existe mais". A história é consciente da existência do passado e quer conhecê-lo, envolvendo crítica, reflexão, distanciamento e objetividade. Sua finalidade é construir uma representação, dentro dos limites impostos pelo passado, ligados às continuidades temporais, às evoluções e as relações das coisas.

As memórias são, portanto, fontes para a história, pois possibilitam saber o que tem sido lembrado por diferentes grupos sociais. Diante dessa perspectiva, seus conteúdos ficam sujeitos a uma análise criteriosa e crítica, pois revelam o que ou quem se quer lembrar e por quê. Assim entendemos que a memória é um fenômeno social plural, admitindo a coexistência de diversas memórias, muitas vezes em disputa pelo que devemos lembrar e pelo que devemos esquecer. Dessa forma, a memória também se refere a relações de poder e é papel do pesquisador investigar os contextos e as condições históricas em que foram produzidas.

O ponto de partida para a construção desse lugar de memória é o trabalho de investigação intitulado "A 'grande família' do Instituto Oswaldo Cruz: a contribuição dos trabalhadores auxiliares dos cientistas no início do século XX". Defendido em 2018 como tese de doutoramento (Reis, 2018), o estudo descreve e analisa os processos, a divisão do trabalho e as relações sociais daí engendradas, buscando apreender a participação dos auxiliares de laboratório dos cientistas do Instituto Oswaldo Cruz na construção da ciência e da saúde pública brasileira entre os anos 1900 e 1930.

A história da Fiocruz está intimamente relacionada ao enfrentamento das epidemias de peste bubônica e febre amarela que assolaram o Rio de Janeiro, então capital da República, no início do século XX. Para aumentar a produção de soro antipestoso em grande escala, foi necessário transferir o Instituto Vacinogênico Municipal para a Fazenda Municipal de Manguinhos, local onde hoje fica localizado o *campus* da Fiocruz no Rio de Janeiro, transformando-o no Instituto Soroterápico Federal, em 1900. Nesse mesmo ano, o médico Oswaldo Cruz é nomeado responsável por toda a parte técnica e pelo serviço de produção de soros.

Dois anos mais tarde, Cruz assume a direção do Instituto Soroterápico, e, em 1903, também a da Diretoria Geral de Saúde Pública, passando a acumular os dois cargos e a participar do plano de intervenção do Estado no saneamento urbano da capital (Fernandes, 1989).

A entrada de Cruz para a diretoria geral facilitou muito a administração do instituto. Foi possível contratar trabalhadores, adquirir novos aparelhos, animais para experiências, livros e revistas científicas do exterior que permitiram a ampliação da biblioteca do Instituto Soroterápico Federal, e a vinda de novos pesquisadores, além da expansão da produção de soros e vacinas para três outras atividades – a fabricação de produtos biológicos, pesquisas médico-experimentais e ensino de bacteriologia (Benchimol, 1990).

Até 1906, as atividades de Manguinhos estiveram mais voltadas para as campanhas sanitárias que ocorriam na capital. As campanhas influenciaram o interesse pelos estudos da microbiologia. Nessa época, foram desenvolvidos os métodos de desinfecção domiciliar de vetores e iniciaram-se as construções monumentais de seu sítio arquitetônico histórico.

No período seguinte, 1906 a 1909, as ações da Diretoria Geral de Saúde Pública ficaram mais restritas à capital, e o Instituto Soroterápico Federal passou a se orientar para ações de profilaxia e estudos de doenças no interior do país, atendendo a demandas do poder público e de empresas particulares, dando origem às grandes expedições científicas do instituto.

Em 1907, com o aval da campanha contra a febre amarela no Rio de Janeiro e após ter obtido o primeiro prêmio na Exposição de Higiene e Demografia em Berlim, Oswaldo Cruz negociou a aprovação de um decreto presidencial que transformou o Instituto Soroterápico em Instituto de Patologia Experimental, rebatizado um ano depois, em 1908, como Instituto Oswaldo Cruz (Benchimol, Teixeira, 1993).

A partir de 1910, inicia-se uma série de expedições ao interior do país, rompendo as fronteiras dos laboratórios, promovendo uma ampliação dos objetos de pesquisa e locais de sua realização, estabelecendo postos permanentes em algumas localidades (Lima, 1998).

Após a criação, em 1918, da Liga Pró-saneamento no Brasil e do Serviço de Profilaxia Rural, o movimento político do sanitarismo campanhista adquire repercussão na imprensa, entre os intelectuais e no Congresso Nacional, culminando com a fundação, em 1920, do Departamento Nacional de Saúde Pública, dirigido por Carlos Chagas, então também diretor do Instituto Oswaldo Cruz (Kropf, Lima, 2010).

A década de 1930 corresponde a acontecimentos históricos como a instituição do governo provisório de Getúlio Vargas e as mudanças estruturais produzidas no âmbito da administração federal, com a criação do Ministério da Educação e Saúde Pública (Mesp) e do Ministério do Trabalho (MT). Nesse contexto, o Instituto Oswaldo Cruz, até então subordinado à pasta da Justiça e Negócios Interiores, é transferido para o Mesp e perde sua autonomia financeira.

A nomenclatura Fundação Oswaldo Cruz e sua abreviatura, Fiocruz, passam a vigorar somente na década de 1970, quando o Instituto Oswaldo Cruz muda sua natureza jurídica para Fundação.

Tendo como lócus principal do trabalho empírico os documentos[1] pertencentes ao Departamento de Arquivo e Documentação da Casa de Oswaldo Cruz/Fiocruz (DAD/COC/Fiocruz), o contato com as fontes possibilitou identificar e analisar aspectos da divisão social do trabalho, a construção de sociabilidades e, em alguns casos, por meio das fontes orais, os percursos de formação profissional e trajetórias de vida.

É importante destacar que os conteúdos sobre os trabalhadores técnicos do Instituto Oswaldo Cruz encontram-se dispersos por todo o acervo, sem que estejam reunidos e sistematizados em um único fundo ou coleção, o que envolveu uma minuciosa e trabalhosa busca por esses auxiliares, que poderiam estar em qualquer lugar do arquivo.

A investigação das fontes contemplou ainda, além dos arquivos do DAD/COC/Fiocruz, a página eletrônica "Memórias do Instituto Oswaldo Cruz" e os portais da Câmara dos Deputados e da Hemeroteca Digital Brasileira.

A partir do cruzamento desse conjunto de fontes foi possível reconstruir aspectos do trabalho dos auxiliares de laboratório em Manguinhos, que não constavam nos documentos oficiais produzidos pela instituição, demonstrando que, desde seu ingresso no instituto, os trabalhadores auxiliares passaram por experiências diversas que produziram uma transformação no seu lugar histórico, pois adquiriram conhecimento, reconhecimento e valor profissional – o que, todavia, não não implicou um rompimento com o lugar institucional subalternizado.

É importante assinalar a ausência de informações mais detalhadas nas fontes consultadas, acerca de dados pessoais desses trabalhadores, como data de nascimento, nacionalidade, raça/cor, escolaridade etc., o que limitou o aprofundamento de análises que seriam fundamentais para compreender aspectos relevantes das relações de trabalho no Instituto Oswaldo Cruz. Também não foi possível obter informações acerca dos efeitos da natureza do trabalho desempenhado pelos auxiliares de laboratório na vida de suas famílias.

É possível afirmar que as relações de trabalho entre os auxiliares de laboratório e os pesquisadores do antigo Instituto Oswaldo Cruz, ao mesmo tempo que colocavam os primeiros em uma situação de submissão vinculada à retribuição do favor e ao apelo pela conquista de laços de pertencimento de família que garantissem um reconhecimento profissional, colaborou para fortalecer, entre os próprios auxiliares, a construção desses laços expressos por meio de práticas de solidariedade e de apoio mútuo.

A seguir, apresentaremos a trajetória de alguns personagens da história do trabalho técnico na Fiocruz, buscando demonstrar as complexas e intrínsecas relações dos auxiliares de laboratório com o Instituto Oswaldo Cruz, que extrapolavam o ambiente de trabalho: era também o local onde moravam, constituíam família, criavam seus filhos, cultivavam amigos e construíam redes de sociabilidades.

## Algumas das muitas histórias de Manguinhos

As histórias retratadas demonstraram que o trabalho desempenhado pelos auxiliares de laboratório do antigo Instituto Oswaldo Cruz foi fundamental para o desenvolvimento da própria Fiocruz, da ciência e da saúde pública brasileira.

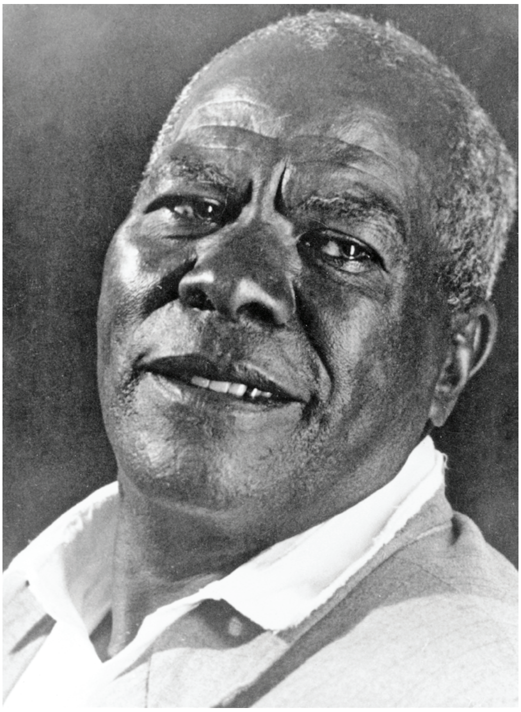

Figura 1: Retrato de Joaquim Venâncio (Retrato..., s.d.)

O patrono da Escola Politécnica, Joaquim Venâncio (Figura 1), um dos mais importantes auxiliares de laboratório da Fiocruz, nasceu em 1895, na Fazenda Bela Vista, uma das propriedades rurais que pertenceram à família do cientista Carlos Chagas, localizada onde hoje é o município de Rio Novo, em Minas Gerais.

Os registros institucionais informam que Joaquim Venâncio chegou ao antigo Instituto Oswaldo Cruz em 1916, com 21 anos de idade. Diversas fontes orais (Gomes, 9 dez. 1985; Oliveira, 8 nov. 2000; Lopes, 3 abr. 1986) corroboram seu vasto conhecimento sobre botânica e zoologia. Além do conhecimento técnico-científico que adquiriu ao longo de sua experiência profissional, já trazia consigo saberes ancestrais e tácitos de sua experiência de vida.

Era reconhecido como "guru", pois tinha prazer em ensinar métodos e técnicas para seus colegas de trabalho. Trabalhou com vários cientistas do antigo Instituto Oswaldo Cruz, e foi citado como colaborador direto em muitos artigos científicos, mas a maior parte do tempo auxiliou diretamente Adolpho Lutz e sua filha, Bertha Lutz.

Quando a cientista estadunidense Doris Cochram, herpetóloga do Smithsonian Institution de Washington, visitou o Brasil na década de 1930 para estudar espécies de anfíbios, foi Joaquim Venâncio que a acompanhou em várias cidades de Minas Gerais e São Paulo. Os estudos e pesquisas de Cochran realizados com a participação de Venâncio foram a base para uma publicação sobre anfíbios do sudeste do Brasil, em 1955, que é uma importante referência para estudiosos dessa área até hoje.

Desde que chegou a Manguinhos, Joaquim Venâncio sempre morou dentro do instituto em uma das casas que pertenciam aos "Próprios Nacionais", imóveis de domínio da União utilizados em serviço público federal, localizados próximo ao Horto Fiocruz.

Foi casado com dona Sebastiana Batista de Carvalho Fernandes, com quem teve cinco filhos: Wanderley, Celso, Joaquim, Renê e Hugo. Os dois últimos foram admitidos no instituto na década de 1940.

Na Figura 2, Joaquim Venâncio aparece trajado com chapéu, paletó e botas, à frente de um grupo de homens ainda sem identificação. Em cima do arbusto está o médico Heráclides de Souza-Araújo, que foi chefe do laboratório de leprologia entre 1927 e 1956. Seus estudos incluíam uma planta medicinal chamada de chaulmoogra, que aparece na foto e, ainda hoje, é muito utilizada pela medicina ayurveda, na Índia, para tratar doenças de pele.

Até os anos 1940 acreditava-se que a chaulmoogra podia curar a hanseníase. Souza-Araújo passou alguns anos, desde 1922, tentando cultivar essa planta no Brasil, sem obter muito êxito, pois o objetivo era a produção do óleo terapêutico. Até que, em 1929, importou quinhentas sementes e as plantou no instituto, algumas no mesmo lugar onde hoje fica o Horto Fiocruz, que era bem próximo da residência de Joaquim Venâncio.

Observando a fotografia, a posição de destaque de Joaquim, como se ele estivesse liderando, conduzindo o grupo até o local onde ficavam as plantas, é provável que Joaquim Venâncio fosse um dos responsáveis, participasse ativamente do cuidado, do cultivo dessa planta em Manguinhos, que chegou a produzir, com êxito, o óleo na década de 1930, distribuído de forma gratuita para centros de tratamento de hanseníase (Santos, Souza, Siani, 2008).

Tanto Joaquim Venâncio como muitos outros auxiliares de laboratório da instituição eram autodidatas que aperfeiçoaram seu ofício no cotidiano do trabalho. Uma das fontes orais consultadas pela pesquisa (Borriello, 13 jun. 1986) revela que inventavam métodos e técnicas próprios, muitas vezes inovando as formas de fazer que aprendiam com os chefes de laboratório.

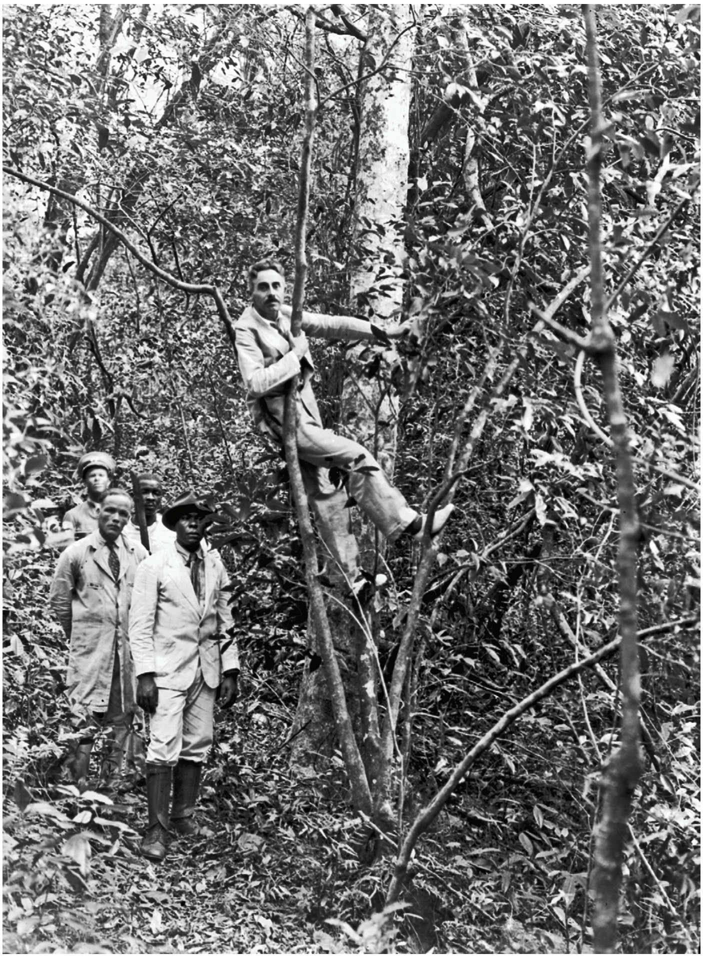

Figura 2: Souza-Araújo, Joaquim Venâncio, e outros trabalhadores não identificados (Souza-Araújo..., 19 maio 1952)

Infelizmente o racismo, a divisão de classe e as relações saber/poder inseridas na totalidade das relações sociais que prevaleciam no período da Primeira República, e que se manifestavam na particularidade das relações de trabalho do antigo Instituto Oswaldo Cruz, reproduziram uma divisão social que limitou o reconhecimento do trabalho e dos saberes dos auxiliares de laboratório.

Nos três primeiros regulamentos da instituição, o pessoal chamado de técnico era constituído pelos cientistas. Os auxiliares eram chamados oficialmente de "subalternos", e os cargos tinham a denominação de "serventes". Os documentos trazem mecanismos explícitos de poder, autoritarismo e distinção, como, por exemplo, a aplicação de penas disciplinares, que poderiam ser verbais ou por escrito, com suspensão de até vinte dias (Reis, 2018).

Essas relações foram mediadas pelo paternalismo, pela lógica do favor e da cordialidade (Chalhoub, 1986; Schwarz, 2000) próprios da gênese da sociedade brasileira, onde os afetos operaram como recursos que buscaram amenizar a exploração do trabalho e os possíveis conflitos emergentes das desigualdades.

Quando observamos as trajetórias profissionais dos auxiliares de laboratório do Instituto Oswaldo Cruz, a primeira questão que se destaca é a presença de ligações de cunho pessoal que atravessam as relações de trabalho na instituição. Elas podiam ser de parentesco, amizade ou algum outro tipo de vínculo, tanto com pesquisadores do instituto como com outros funcionários.

A personalização implica o entrelaçamento de um compromisso que submete ambas as partes a uma contraprestação de serviços: de um lado a identificação do subalterno com os interesses do mais poderoso, de outro a oferta de proteção e de pequenas benevolências, que dissimulam os excessos da autoridade de seu superior. Essa autoridade era considerada essencial para que o trabalhador fosse compelido a desempenhar suas tarefas da forma mais eficiente possível (Chalhoub, 1986).

As relações pessoais são pressupostos para que se estabeleça uma ideologia do favor (Schwarz, 2000). Este se constitui como um dos mecanismos da mediação capital *versus* trabalho, que busca disfarçar e amenizar a violência gerada pela desigualdade presente nessa relação, em que uma das partes, a não proprietária, está sempre em desvantagem.

Considerando o período histórico da pesquisa, concordamos com a análise de Schwarz ao afirmar que o favor atua entre o escravismo e as ideias liberais, encontrando um meio-termo. Uma maneira de mudar as relações sem as transformar em sua essência. Dessa forma o mecanismo do favor, que em princípio é incompatível com as ideias liberais republicanas, as absorve e transforma seu sentido atuando por meio de uma relação que pressupõe estima, afetos, símbolos e significados e que, em última análise, pratica e conserva as relações de dependência entre patrões e empregados.

Essas mediações estiveram presentes desde o recrutamento e a seleção desses trabalhadores, passando pela moradia no local de trabalho, por sua ascensão funcional, nas estratégias de transpor o acesso ao conhecimento científico e na relação com os cientistas.

O favor traspassa o conjunto da existência nacional, mediando as relações de trabalho nos mais variados campos e profissões e é potencializado pelos preceitos liberais da igualdade e do mérito. Dessa forma, todos dependem dele: os profissionais para exercer sua profissão, os pequenos proprietários para garantir a segurança de sua propriedade e o funcionário para garantir seu posto de trabalho (Schwarz, 2000).

Nessa lógica de criação de laços de dependência, os trabalhadores subalternizados são gratos aos seus superiores, pois é por causa deles que mantêm seu emprego e sua subsistência. A contrapartida dessa gratidão é a expectativa de um "bom comportamento" no trabalho: leal, fiel, dedicado, que executa suas tarefas com amizade, com amor.

A ideologia do favor esteve presente em muitos aspectos das relações de trabalho do Instituto Oswaldo Cruz. A oportunidade de ingresso no instituto foi atravessada pelo entrelaçamento de um comprometimento, que submetia o requerente ao posto de trabalho à chancela do empregador. Principalmente porque no processo seletivo havia ausência de critérios claros de recrutamento e das atribuições necessárias ao cargo. Eram trabalhadores com pouca ou quase nenhuma escolaridade, prevalecendo no momento do pleito a oferta de um trabalho sem qualificação.

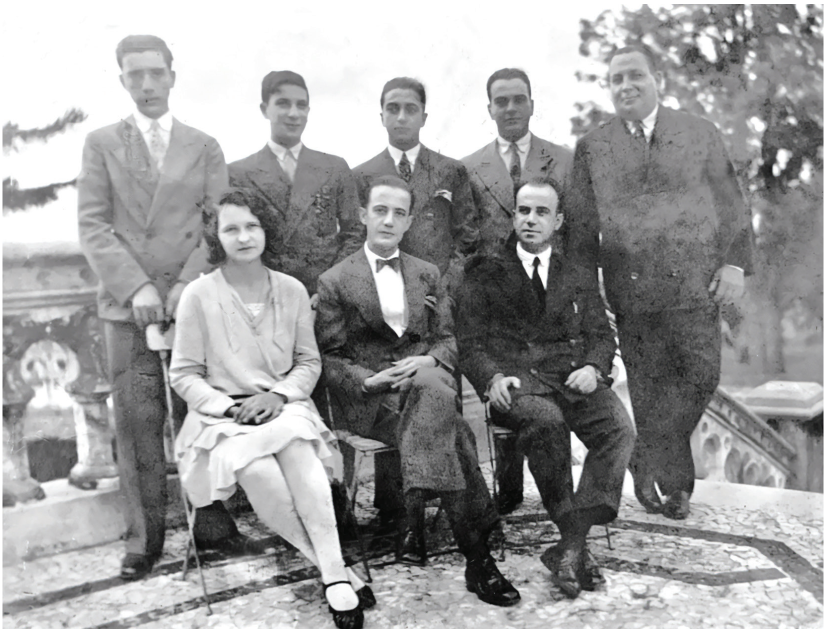

Figura 3: Família Borriello, no dia do casamento de Attilio com Anna da Cunha. Da esquerda para a direita, em pé: Miguel Marcondes, sobrinho de Attilio, os irmãos Salvador, José e Benedito, e um amigo da família, sem identificação. Sentados: Anna da Cunha Borriello, Attilio Borriello e o irmão mais velho, Antonio (Acervo pessoal da família Borriello)

A Figura 3 foi feita no *campus* da Fiocruz, mais precisamente no acesso às escadarias do prédio do Castelo, no dia do casamento do senhor Attilio Romulo Borriello com a senhora Anna da Cunha.

A família Borriello era da mesma cidade onde nasceu Oswaldo Cruz, São Luiz do Paraitinga, no interior de São Paulo. O irmão mais velho, Antonio Borriello, era amigo de Waldomiro Rodrigues de Andrade, tesoureiro do instituto, que, por sua vez, era amigo do pai de Oswaldo Cruz. Waldomiro arranjou um trabalho no almoxarifado para Antonio, que posteriormente trouxe os irmãos, Benedito, José, Salvador e Attilio.

Os Borriello moravam em uma casa localizada em uma das colinas do *campus* e demolida em 1935 para dar lugar ao edifício construído pela Fundação Rockefeller. O espaço era compartilhado com mais duas outras famílias de auxiliares de laboratório: a de José Joaquim Dias Paredes, encarregado da seção de meios de cultura, e suas filhas; e a de José de Vasconcellos, com sua esposa e três filhos. Atualmente, nesse prédio, funcionam alguns laboratórios da unidade técnico-científica de Biomanguinhos.

Attilio Borriello veio para o antigo Instituto Oswaldo Cruz com 15 anos, em 1921. Começou trabalhando na tipografia e logo depois foi transferido para o laboratório de protozoologia. Nos anos 1930 foi auxiliar do próprio Carlos Chagas, na época diretor do instituto, participando dos estudos sobre o *Trypanosoma cruzi*.

Agindo como sujeitos de suas próprias histórias, os auxiliares de laboratório souberam transitar pelos meandros de uma hegemonia cultural da instituição, que tentava impor um modo de vida em que o cientista era o soberano. Assim, atuaram de forma a reverter os mesmos mecanismos de manutenção da hegemonia dominante em favor de seus próprios interesses, como, por exemplo, no acesso aos livros da biblioteca, vedados a eles, e na colocação de seus filhos e outros familiares em postos de trabalho no instituto.

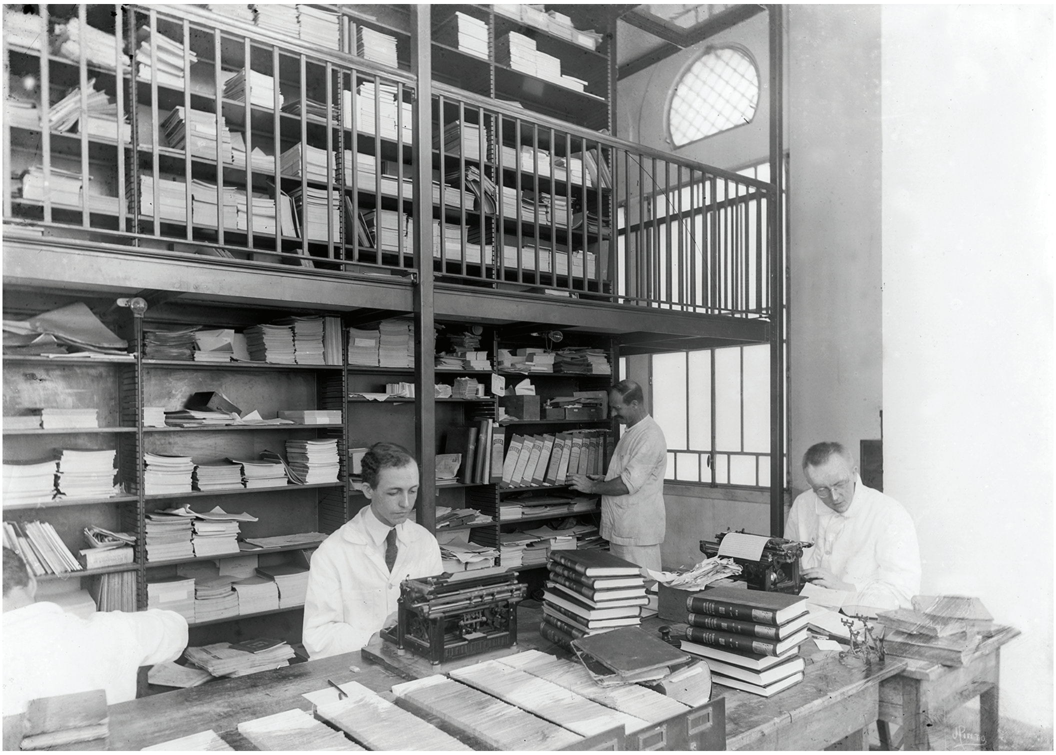

Figura 4: João Simões Paulo, junto às estantes da biblioteca. Assuerus Overmeer sentado à cabeceira da mesa (Assuerus..., s.d.)

A Figura 4 retrata os funcionários da biblioteca exercendo suas atividades. Sentados à mesa, diante das máquinas de escrever, estão Assuerus Overmeer, de óculos, chefe da biblioteca, e outro trabalhador sem identificação. Ao fundo, organizando a estante, está João Simões Paulo, mais conhecido como "João o Grande" (Fonseca Filho, 1974).

João o Grande ingressou no instituto trabalhando nas obras dos edifícios que hoje pertencem ao Nahm. Era muito amigo e vizinho do mestre de obras, Basílio Aor, e foi padrinho de um de seus filhos. Depois que as obras terminaram, João Simões foi transferido para trabalhar na biblioteca, e como era ele quem cuidava das fichas, limpava e arrumava os livros, de vez em quando emprestava um exemplar para seus colegas auxiliares de laboratório, escondido dos cientistas.

A moradia no instituto, ao mesmo tempo que os submetia a um regime de trabalho quase ininterrupto, favoreceu relações de solidariedade e ajuda mútua entre os companheiros de trabalho, como a organização de práticas associativas que envolveram sociabilidades de diferentes ordens.

Uma delas, o futebol, era uma paixão compartilhada pelos auxiliares de laboratório do antigo Instituto Oswaldo Cruz, tanto assim que, em 2 de dezembro de 1915, fundaram, por iniciativa própria, o Manguinhos Futebol Clube, que tinha como princípio o desenvolvimento atlético de seus associados. O time chegou a disputar vários campeonatos na década de 1920, consagrando-se campeão da Liga Brasileira de Desportos em 1921. Seu uniforme tricolor trazia as cores vermelho, preto e branco.

Da mesma forma, a espiritualidade reuniu um grupo de auxiliares de laboratório e trabalhadores das oficinas[2] que compunham as seções administrativas do antigo Instituto Oswaldo Cruz, fundando, em 1933, uma entidade religiosa beneficente, a Congregação Espírita Oswaldo Cruz,[3] que continua em funcionamento até hoje, no bairro de Bonsucesso.

O acesso à educação formal, limitado pela condição social dos trabalhadores auxiliares, não impediu que adquirissem os conhecimentos necessários para o bom desempenho de seu trabalho. O aprendizado com os cientistas e com os colegas de laboratório no instituto proporcionou ascensão profissional por dentro da classe, mas não chegou a representar uma ruptura de seu lugar subalterno na instituição.

Os limites dessa ascensão restringiram-se em torno de algumas prerrogativas que exprimiam o que se esperava de um bom auxiliar de laboratório: o respeito à hierarquia, a dedicação, o amor e a lealdade. Premidos por esse horizonte identitário, importava para esses trabalhadores chegar a ser um técnico completo, um auxiliar dileto e conseguir um posto de trabalho para seus familiares.

## Um lugar de memória para os trabalhadores técnicos da Fiocruz

Em 2020, em plena pandemia de covid-19, a Fiocruz comemorou seu aniversário de 120 anos. Entre as muitas celebrações que foram possíveis naquele contexto, o lançamento do edital para apresentação de propostas para Projetos de Memória Institucional compunha um conjunto de iniciativas promovido pela Casa de Oswaldo Cruz e apoiado pela Vice-presidência de Educação, Informação e Comunicação da Fiocruz.

O projeto "Lugar de memória: história e vida dos trabalhadores técnicos da Fiocruz" foi uma das propostas contempladas pelo edital.

O propósito do projeto foi constituir um local de memória para os trabalhadores técnicos em saúde da Fundação Oswaldo Cruz, de forma a contribuir para a construção e preservação de uma memória coletiva, considerando as experiências históricas vividas por esse grupo de trabalhadores e dando visibilidade a outras narrativas que fazem parte da trajetória da instituição.

Nesse sentido, o projeto pretendeu estabelecer um lugar de materialidade para que a memória desses trabalhadores possa ser apreendida por toda a comunidade Fiocruz, funcionando como alicerces de uma memória coletiva que pode revelar e expressar o compartilhamento de identidades comuns.

Seu objetivo buscou, de maneira geral, contribuir para a preservação da memória do trabalho e dos trabalhadores técnicos em saúde da Fiocruz nas distintas temporalidades da história, considerando suas possibilidades de diálogo com o tempo presente e as diversas pesquisas, experiências, narrativas, testemunhos e trajetórias desses trabalhadores na instituição em seus 120 anos de existência.

O produto desse projeto foi a criação de um lugar de memória que abrigasse a história dos trabalhadores técnicos da Fiocruz em sua gênese, funcionando ao mesmo tempo como um lócus de preservação do patrimônio imaterial do trabalho técnico em saúde da instituição, um ambiente pedagógico de conhecimento e um repositório de fontes para pesquisas sobre o tema que possa colaborar para fortalecer o sentimento de pertencimento à Fiocruz entre seus estudantes, funcionários, docentes e pesquisadores.

Assim, a ideia foi criar um ambiente virtual dinâmico em contínua ampliação e atualização sobre a história do trabalho e dos trabalhadores técnicos da Fiocruz, atualizado de acordo com o andamento de estudos alinhados à temática.

O *site* da exposição está disponível no servidor da Escola Politécnica de Saúde Joaquim Venâncio.[4] Ao acessá-la, o público poderá conhecer a história dos trabalhadores técnicos da Fiocruz, por meio de pequenos textos elaborados especialmente para esse ambiente, acompanhados por objetos expográficos (fotografias, documentos digitalizados, áudios, vídeos etc.).

O *site* está organizado em oito módulos ou espaços expositivos que abordam aspectos da vida e do trabalho dos auxiliares de laboratório da Fiocruz em seus trinta primeiros anos de existência: Panorama (breve contextualização histórica do mundo do trabalho nos primeiros anos da República brasileira, a ideologia moralizante do trabalho no Brasil e seus reflexos em Manguinhos); Biografias (minibiografia de 22 auxiliares de laboratório que atuaram no IOC nas três primeiras décadas do século XX); Trabalho (aspectos do trabalho dos auxiliares no IOC: que tipos de trabalho faziam; formas de ingresso; ascensão funcional; penalidades e punições; relações com seus superiores e suas muitas contradições; aspectos sociais da divisão do trabalho – hierarquias, racismo, divisão de classes); Educação (os aprendizados no trabalho com os cientistas e com os colegas; acesso à educação formal; saberes tácitos e saberes vivenciais); Moradia (locais onde moravam nos terrenos do Instituto Oswaldo Cruz; relação trabalho e moradia; construção do muro depois dos anos 1930); Expedições (o trabalho dos técnicos nas expedições científicas; identificação dos auxiliares que participaram de expedições nas décadas iniciais do século XX; auxiliares da expedição Penna e Neiva); (Re)existências (as redes de sociabilidades criadas pelos técnicos; companheirismos e solidariedade; mecanismos de (re)existências e insubordinações; política, lazer, esporte, religiosidade); Legados (espaço que pretende interagir com o público por meio de relatos, testemunhos e depoimentos de ex-alunos da EPSJV, de familiares de antigos técnicos, moradores da comunidade do entorno da Fiocruz e muitos outros).

Na página inicial, é possível acessar uma seção com conteúdos educativos, organizados em um mural virtual (feito com a ferramenta Padlet), para aqueles visitantes que desejarem saber mais sobre alguns dos temas abordados na exposição e o jogo Fiocraft, projeto que reproduz uma versão virtual da instituição dentro de um dos jogos digitais mais populares do mundo, o Minecraft. As placas com os pontos de memória estão reproduzidas também dentro do jogo, que foi desenvolvido por uma equipe de pesquisadores do Instituto de Comunicação e Informação Científica e Tecnológica (Icict) e da Escola Politécnica de Saúde Joaquim Venâncio (EPSJV).

Além do *site*, o projeto estabeleceu o que chamamos de pontos de memória em vários locais do *campus* da Fiocruz no Rio de Janeiro, inicialmente nos prédios que compõem o conjunto do Nahm, e na própria EPSJV.

> O Nahm é o núcleo originário do *campus* formado pelas edificações erguidas nas duas primeiras décadas do século XX: o Pavilhão Mourisco, o Pavilhão do Relógio, a Cavalariça, o Pavilhão Figueiredo de Vasconcelos, o Pombal, a Casa de Chá, o Hospital Evandro Chagas e o Pavilhão Vacínico, hoje denominado Vila Residencial. O plano de requalificação em questão abrange as edificações deste núcleo originário, à exceção do Hospital Evandro Chagas e da Vila Residencial, incluindo também a Praça Pasteur, o Caminho Oswaldo Cruz e o Pavilhão Henrique Aragão, de modo a conformar uma área contínua de intervenção (Fundação..., 2014).

Os pontos de memória estão identificados por placas instaladas em frente à entrada principal das edificações, contendo um texto interpretativo com informações históricas sobre o prédio, relacionando-as às histórias dos auxiliares de laboratório que ali viviam ou trabalhavam. As placas têm um código QR que, quando acionado com a câmera de um celular, encaminha o visitante ou o trabalhador da instituição para o *site* da exposição virtual.

A ideia é que, após a conclusão do Projeto de Requalificação do *Campus*, as placas iniciais sejam substituídas, acompanhando a identidade visual proposta para a sinalização do *campus*, e sejam instaladas mais seis placas em outros locais relevantes para a história dos técnicos da Fiocruz, o Pombal, o Pavilhão Rockefeller e demais.

As localizações das placas estão reproduzidas na página inicial do *site* por meio de um mapa interativo do *campus* da Fiocruz. Cada símbolo de localização, quando acionado, mostra uma foto dos edifícios e reproduz parte do texto interpretativo.

O caráter dinâmico do *site* tem como intenção a possibilidade de agregar, em uma perspectiva futura, novas pesquisas realizadas sobre os trabalhadores técnicos da Fiocruz, deste e de outros períodos cronológicos de sua história, para compor a exposição. Assim como sua integração a outros ambientes digitais previstos em estudos e pesquisas da EPSJV, de outras unidades técnico-científicas ou instituições de saúde, que guardem afinidades com o tema, buscando estabelecer um diálogo com a história do presente, por meio de memórias, narrativas, testemunhos e trajetórias de trabalhadores da saúde.

## Considerações finais

A história dos auxiliares de laboratório da Fiocruz fala de pessoas comuns, ordinárias. Não estamos tratando de grandes homens, de heróis ou mitos. Para muita gente essas histórias podem parecer irrelevantes, causar pouca curiosidade ou interesse. Ao mesmo tempo são histórias fragmentadas, constituídas de muitas ausências e silêncios, típico das classes subalternizadas (Gramsci, 2002).

São, no entanto, histórias de enorme relevância para pensar o campo da Educação Profissional em Saúde. Compreender os muitos aspectos dessa memória, história, a divisão do trabalho, a divisão de classes, as disputas que perpassam a reprodução social da existência, é fundamental para que possamos refletir sobre quem são os trabalhadores técnicos da saúde hoje e como podemos colaborar para que seus processos educativos sejam concebidos como formação humana, superando as dicotomias que reduzem a oferta da educação profissional a meros treinamentos, reservando para os trabalhadores técnicos da saúde um aprendizado que está sempre subalternizado em relação aos profissionais de nível superior.

Além disso, são as nossas histórias, histórias que nos constituem como comunidade e que trazem outros ângulos para que possamos conhecer e refletir sobre a trajetória da Fiocruz em suas três primeiras décadas de existência, buscando fortalecer um sentimento de identificação e de pertencimento dos atuais trabalhadores técnicos da instituição com aqueles que os precederam no passado.

Nessa perspectiva, concordamos com Walter Benjamin (2012, p.249) quando assevera que o passado contém o presente, e o lugar da história é preenchido de um "tempo de agora". O autor propõe uma forma de produção da história por meio de uma reflexão sobre a recuperação e reconstrução do passado em sua relação com o presente, o que significa também a junção entre história e política e entre rememoração e redenção: "A imagem da felicidade está indissoluvelmente ligada à da redenção. O mesmo ocorre com a representação do passado que a história transforma em seu objeto" (p.242).

A rememoração é a possibilidade de revisitar o passado e realizar uma reparação, seja individual ou coletiva, no campo da história, evocando as experiências das gerações que nos precederam para então as redimir, e assim também nos redimir, buscando "... uma transformação ativa do presente" (Gagnebin, 2013, p.105).

Aqui o caráter messiânico é, na verdade, emancipatório: não há um messias, nós somos os agentes da nossa libertação. Nessa perspectiva, apropriar-se historicamente do passado significa apropriar-se de uma recordação, do modo de seu relampejar no momento do perigo. O perigo a que o autor se refere é o do conformismo diante da história, apontando para a possibilidade de subvertê-la, tornando aparente suas contradições. O papel do historiador é o de despertar no passado a luta pelos aspectos brutos e materiais, sem os quais não existem os refinados e espirituais, o que implica contar a história considerando os elementos espirituais que se relacionam com a cultura e que Benjamin (2012) define como confiança, coragem, humor, astúcia e firmeza, que vão constituir um sentimento de classe.

A criação desse Lugar de Memória vem ao encontro do sentido que Benjamin atribui, rememorando e redimindo o trabalhador técnico da Fiocruz do esquecimento e do silêncio sobre suas narrativas e trajetórias na instituição, oferecendo às novas gerações uma reflexão sobre os caminhos trilhados e novas possibilidades de percurso na construção da história institucional.

AGRADECIMENTOS

O artigo é produto do projeto "Lugares de memória: história e vida dos trabalhadores técnicos da Fiocruz", contemplado com financiamento da Fiocruz para Projetos de Memória Institucional (chamada interna 2020).

## NOTAS

[1] As fontes exploradas nos arquivos do DAD/COC/Fiocruz contemplaram os Livros de Registros pertencentes à Seção Cadastro de Funcionários Estatutários, documentos administrativos, técnico-científicos, correspondências, relatórios, ordens de serviço, documentação sobre os imóveis construídos nos terrenos do instituto e que serviam de moradia para seus funcionários, depoimentos orais do Projeto Memória de Manguinhos e a coleção de imagens do acervo da Casa de Oswaldo Cruz.

[2] As seções administrativas compunham-se de secretaria, zeladoria, almoxarifado e arquivo, e as seções auxiliares eram compostas por biblioteca, museu, desenho, fotografia e microfotografia, tipografia, esterilização e preparo de meios de cultura, distribuição de soros e vacinas, biotérios e cavalariças, mecânica e eletricidade, carpintaria, conservação de imóveis e estradas, oficinas de encadernação, de preparação de ampolas e de aparelhos de vidro.

[3] A Congregação Espírita Oswaldo Cruz foi fundada no dia 25 de março de 1933, por iniciativa de Abílio Lopes de Oliveira, auxiliar de laboratório que ingressou em Manguinhos em 1913.

[4] Endereço eletrônico para acesso: www.expomemorias.epsjv.fiocruz.br.
